# Assessment of Supply and Demand of Regional Flood Regulation Ecosystem Services and Zoning Management in Response to Flood Disasters: A Case Study of Fujian Delta

**DOI:** 10.3390/ijerph20010589

**Published:** 2022-12-29

**Authors:** Jian Tian, Suiping Zeng, Jian Zeng, Feiyang Jiang

**Affiliations:** 1 School of Architecture, Tianjin University, Tianjin 300072, China; 2 School of Architecture and Urban Planning, Tongji University, Shanghai 200092, China; 3 School of Architecture, Tianjin Chengjian University, Tianjin 300384, China

**Keywords:** flood regulation, ecosystem service, social–ecological system, flood hazard risk, Fujian Delta, across spatial scales, zoning management

## Abstract

Global climate change has led to flood disasters increasing in terms of frequency and damage caused, which seriously threatens urban and rural security. The flood regulation (FR) service function of the ecosystem plays an important role in mitigating flood disaster risk. Previous studies on flood regulation ecosystem services (FRES) are still lacking in a cross-scale assessment of supply and demand, refined simulation of regional complex hydrology, and application of spatial zoning management. Taking the Fujian Delta as an example, this study established a cross-scale research framework based on the social-ecosystem principle. The SWAT model was used to simulate the regional hydrological runoff and calculate the macro-scale supply of FRES. Taking patches of land as units, a flood risk assessment model was constructed to calculate the micro-scale demand for FRES for urban and rural society. Through a comparison of supply and demand across spatial scales, a zoning management scheme to deal with flood disaster risk was proposed. The results showed that: (1) The supply of FRES differed greatly among the sub-basins, and the sub-basins with low supply were mostly distributed in the lower reaches of Jiulong River and the coastal areas. (2) The demand for FRES was concentrated in high-density urban built-up areas. (3) By comparing the supply and demand of FRES in sub-basin units, 2153 km^2^ ecological space was identified as the primary ecological protection area, and 914 km^2^ cultivated land and bare land were identified as the primary ecological restoration area. (4) By comparing the supply and demand of FRES of land patch units, 65.42 km^2^ of construction land was identified as the primary intervention area. This study provides a decision-making basis for regional flood disaster management from the perspective of FRES.

## 1. Introduction

Global warming has led to frequent extreme rainfall, and floods have become one of the major disasters threatening the security of urban and rural human settlements [1,2]. In 2020, the frequency of global flood disasters accounted for 61.66% of the total annual frequency of natural disasters [3]. With the rapid development of urbanization, the demand for flood regulation (FR) service in urban and rural areas is constantly increasing [4,5]. Only relying on engineering measures to deal with flood disaster risk will lead to huge investment and a limited scope of regulation [6,7,8]. It is increasingly recognized that ecosystems can play an important role in regulating surface runoff and reducing the risk of rainstorms and floods [1,9]. Ecosystem services (ES) are the conditions and processes that maintain and satisfy human life [10,11]. The supply of ES are the products provided by the ecosystem for human welfare, and the demand for ES is the consumption of the products provided by the ecosystem [11]. Ecological and environmental problems are fundamentally caused by imbalances in the supply and demand of ES [11]. The flood regulation ecosystem services (FRES) is an important part of ES [1,6]. It reflects an ecosystems’ ability to reduce flood hazards caused by heavy precipitation and upstream discharge and is mainly determined by surface characteristics such as soil, land use, and vegetation cover [12,13]. Recently, the research on flood risk management based on FRES has become a hot topic for scholars in related fields [12].

Existing studies have made abundant achievements in the analysis of the FRES mechanism [12,13,14], the identification of key flood control areas based on ES [8,15,16,17], and the changes in the supply and demand of FRES during urbanization [2,4,5,16]. In view of the shortcomings of existing achievements in cross-spatial scale comparisons of supply and demand, a refined simulation of the regional complex hydrological environment and the application of spatial zoning management, the research on the supply and demand of FRES needs to be further explored and, specifically, the following three aspects:

Firstly, it is necessary to strengthen the research on the supply and demand of FRES across spatial scales. In recent years, the comparison of FRES supply and demand has attracted extensive attention from many disciplines [6,12,18], but the spatial scale of the comparisons are relatively few. Among them, studies at the macro-watershed scale usually take the sub-basin as the spatial unit, which is conducive to scientific simulations of regional surface runoff level [1,6], but makes it difficult to reflect the difference in flood susceptibility or risk among micro-spatial units [19]. Micro-urban scale studies, which usually take blocks or land patches as spatial units, are conducive to accurately identifying flood susceptibility [8,15,16,20], but ignore the hydrological regulation mechanism of macro-regional ecosystems [5,6], which makes it difficult to accurately calculate the supply capacity of FRES. The supply of FRES is formed by watershed in regional environments [12,21,22]. The FRES demands of human society have micro-differences due to the different population, economy and facilities of each land-patch unit [19]. Using the concept of “service spatial flow” [19] to link sub-basin units with land-patch units and compare them across spatial scales is an effective way of solving this problem.

Second, it is necessary to improve accuracy when measuring FRES supply and demand. Most of the existing measurement methods of FRES supply are calculated by hydrological environment simulation [5,6], for example, the InVEST model [10,23], SCS-CN hydrological model [5,16,24,25,26,27], STREAM model [6], runoff curve number (RCN) approach [14], hydrological model (CLM-GBHM) [21], ARIES model method [28]. However, the above calculation methods and simulation models cannot consider the complexity of a regional hydrological environment. The regulation of ecosystems prone to flooding is influenced by the regulation of the wandering watershed in the region and the combined effect of complex and variable climate, hydrology, soil, land use, vegetation cover, and plant transpiration [1,22]. Therefore, a surface runoff simulation and calibration model should be adopted to simulate the real hydrological formation process in a complex environment, and more detailed climate, soil, land-use databases, and calibration methods should be used to improve the assessment accuracy of FRES supply [9,22,29]. Current methods of measuring the demand for FRES focus on drawing susceptibility or risk zoning maps [30,31,32,33] through flood susceptibility or risk analysis [17,34,35,36]. Specifically, this includes economic loss calculation [6], inundated area simulation measurements [15], the index weight method [18], data mining and machine learning [30,31], the logistic regression model [33,37], the analytic hierarchy process [17,32,35], frequency ratio method [36] and the deep neural network method [38,39]. The assessment factors mainly involve the risk of disaster-causing factors, the sensitivity of the disaster-bearing environment and the vulnerability of disaster-bearing bodies [15,39], but less consideration has been given to disaster reduction and the relief facilities of disaster-bearing bodies. Weak disaster reduction and relief capacity will increase flood susceptibility [34] and increase the demand for FRES.

Thirdly, the application level of the research results for the spatial zoning management of flood risk should be improved. At present, the matrix method [20], value equivalent method [40], spatial autocorrelation method [4] and data space superposition method [41] are usually used to compare the supply and demand of FRES, identify the regions with an imbalance in FRES supply and demand, and divide the intervention zones of flood control projects [8,15]. However, the spatial zoning management of flood risk should not be limited to interventions in flood control projects in urban areas. The protection and restoration of regional ecological space is of great importance to improve FRES’ abilities and reduce the risk of flood disasters [1,40]. Therefore, it is necessary to finely divide ecological space protection and restoration zones based on the comparison results of the supply and demand of FRES.

Based on the above analysis, we propose the following solutions: (1) Regarding the comparison of FRES supply and demand, the concept of “service spatial flow” [19] is introduced to realize cross-spatial comparison by pooling the FRES demands of land patches to sub-basin units and by decommitting the FRES supply of sub-basins to land-patch units. (2) In terms of the regional hydrological simulation, the SWAT model and the SWAT-CUP calibration model are adopted to simulate the real hydrological formation process in complex environments [9,22]. In terms of storm flood risk assessment factors, the adaptability indexes of disaster-bearing bodies, reflecting disaster reduction and relief ability, should be added to improve the accuracy of FRES demand measurements. (3) In terms of the application of the results, by superimposing the spatial units of the imbalance between supply and demand and land-use data, the zoning of ecological space protection and restoration and the zoning of flood control intervention in built-up areas are proposed. From the two dimensions of increasing supply and reducing demand, spatial zoning management can be formed to deal with flood risks and strengthen the connection with spatial management.

## 2. Study Area and Data Sources

### 2.1. Study Area

**Figure 1:**
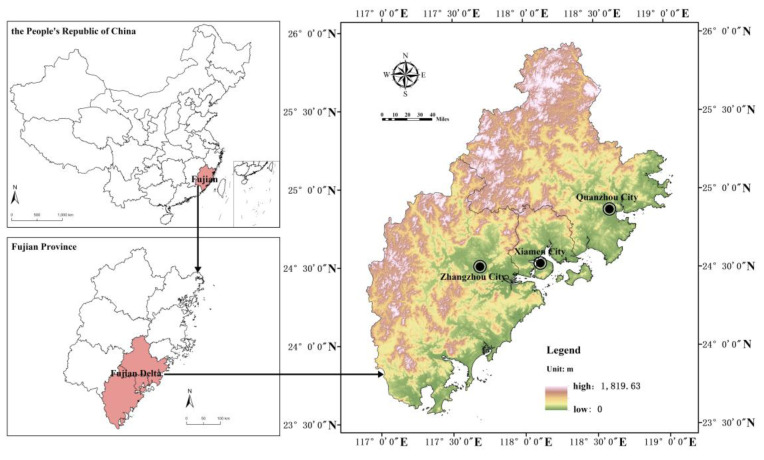
Location and topography of Fujian Delta.

Fujian Delta is located in the southeast coastal area of Fujian Province, China, including Xiamen, Quanzhou and Zhangzhou, which had a total area of 25,323 km^2^ and a permanent resident population of 19 million in 2020. The northwest is dominated by mountains, while the southeast is coastal hilly landform, with the highest altitude of 1819.63 m. The mountains and seas are staggered, and the terrain varies greatly (Figure 1). Affected by the terrain, the drainage basin area in the Fujian Delta is small, the river flow is short, the flow rate is fast, and the flow is large. Under the action of tropical cyclones, the extreme weather conditions of typhoon and rainstorm in summer are frequent, the rainfall is large, and the river flood season is long. Due to the dense distribution of towns and high population density in coastal areas, urban and rural society is more seriously threatened by flood. Therefore, it is urgent to optimize the regional ecological spatial pattern, improve the capacity of FRES, and reduce the risk of flood damage.

### 2.2. Data Sources

This study uses an extensive dataset covering DEM, land use, meteorology, soil type, NDVI, roads, and population to measure the supply and demand of FRES. Our data mainly derive from official open sources and a series of satellite images. For example, the normalized difference vegetation index (NDVI) was calculated using Landsat 8 remote sensing images. Based on a geospatial data cloud, DEM elevation data were obtained, and the slope was further calculated by GIS [24]. Land-use data were obtained based on the Data Center for Resources and Environmental Sciences, Chinese Academy of Sciences. According to the data requirements of the SWAT model, soil-type data were obtained based on HWSD soil database, and meteorological data were obtained based on CMADS dataset [29,42]. The data on water system, road, medical treatment, and firefighting facilities were downloaded based on AMAP and the universal electronic map of Shuijingzhu. In addition, public data were obtained from relevant institutions (such as hydrological observation data provided by a hydrological station in Zhangzhou, Fujian province). More detailed data sources are shown in Table 1.

## 3. Methodology

### 3.1. A Cross-Scale Framework for FRES

#### 3.1.1. Supply and Demand of FRES from a Socio-Ecosystem Perspective

The socio-ecological system is composed of human society and the living environment [19]. Under heavy rainfall, areas with a sensitive disaster environment are prone to producing strong floods, which have a strong impact on human society. By regulating surface runoff, natural ecosystems can effectively alleviate the risk of flood damage of disaster-bearing bodies [12]. Among them, the supply of FRES is affected by the comprehensive effect of macro-regional topography, climate, vegetation, hydrology and soil conditions [12,21,22], forming a stormwater regulation mechanism with sub-basins as spatial units [6,9]. Due to the different densities of populations, industries, construction, disaster reduction and rescue facilities, the FRES demand of human society shows obvious micro-differences in different land patches [16,19]. Therefore, it is necessary to coordinate the spatial relationship between human society’s demand for disaster prevention and the supply of ES by comparing the supply and demand of FRES across spatial scales, to provide a decision-making basis for the zoning management of flood risk.

The term sub-basin refers to dividing the region into several catchment units according to watershed and catchment characteristics for the convenience of detailed research. In this study, the sub-basin is the catchment unit that forms surface runoff, and it is also the basic analysis unit of surface runoff simulation in SWAT model to study the impact of the ecosystem on surface runoff and flood regulation. The size of the sub-basin depends on the specific research needs. In this study, the area of each sub-basin was no less than 50 km^2^. Land patch is the smallest spatial unit used to analyze the spatial heterogeneity of flood disaster risk in this study. There are many ways to divide land patches, and this study uses 100 m × 100 m grid units.

#### 3.1.2. A Cross-Scale Framework for FRES Based on the Service Spatial Flow

**Figure 2:**
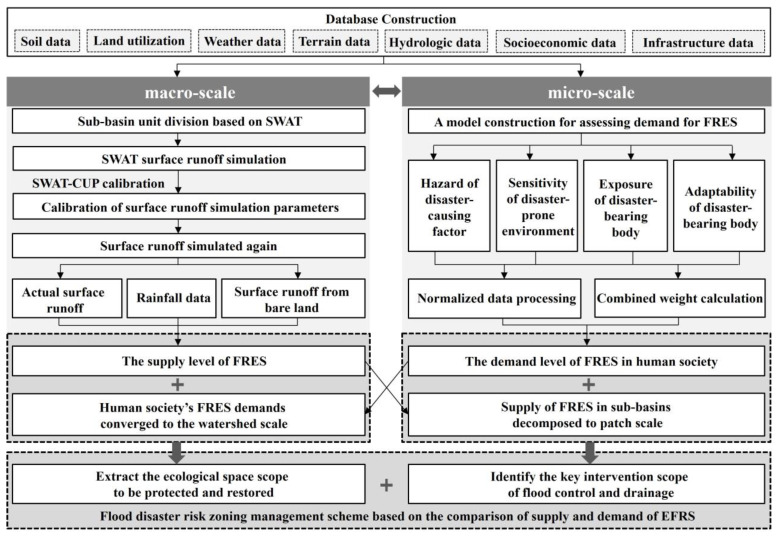
Research framework and technical route.

Based on the concept of service spatial flow, cross-scale comparisons are possible. That is, in the watershed space, the FRES demands of the microscopic land patches are pooled into the sub-basin unit, and the FRES supply of the macroscopic sub-basin unit is decomposed into the land patches, to realize the cross-scale comparison of supply and demand. Therefore, the technical route of this research is shown in Figure 2. Firstly, the catchment unit of the molecular watershed was delimited, the surface runoff was simulated based on the SWAT model [9,22], and the supply of FRES was calculated by taking the sub-basin as the unit. Secondly, based on the risk assessment principle of “disaster-causing factor, disaster-prone environment, disaster-bearing body” [15,39], a FRES demand assessment model for human society was constructed, and the demand for FRES was calculated by taking land patches as units. Thirdly, the supply and demand of FRES were compared across spatial scales. By pooling the FRES demand of each land patch into the sub-basin, the ecological space of the sub-basin with high regulation demand was mainly protected and restored. Through the decomposition of the FRES supply of sub-basin ecosystems to land patches, the construction land patches that urgently require manual intervention to reduce the risk of stormwater can be identified. Finally, a flood disaster risk-zoning management scheme is proposed based on the comparison of FRES supply and demand.

### 3.2. Assessment Methods for the Supply of FRES

#### 3.2.1. Simulation and Verification of Surface Runoff Based on SWAT

The SWAT model was selected to simulate and rate surface runoff. SWAT is a distributed watershed hydrological model based on the GIS platform which is mainly used to predict the impact of hydrology, sediment, and chemical substances in the flow domain of land-use planning [9]. Its simulation object is a watershed scale, which can simulate hydrology and related material migration and transformation by integrating the topography and geology, soil, land use, weather, and management measures of the basin. The SWAT model can calculate hundreds of sub-basins at the same time and has the characteristics of a flexible watershed and sub-basin and calculation structure [29]. At present, the model has become an indispensable tool in water conservation management planning and is used by some national and local government decision makers. This study mainly applied the surface runoff simulation function of this model, which can accurately simulate surface runoff by comprehensively processing refined and complex data such as topography, soil, land use, meteorology, and hydrology [22] to provide a basis for calculating the supply capacity of FRES.

SWAT-CUP is a connector program that connects optimization programs with SWAT to conduct sensitivity and uncertainty analyses of the SWAT model on behalf of users, and realize model calibration and verification. The program considers all sources of uncertainty, and the degree of uncertainty is measured using P-factor (95% confidence interval contains the proportion of measured values) and R-factor (95% average width of the uncertainty interval). After obtaining the appropriate P-factor and R-factor, the goodness of fit was further quantified by R^2^ and NS between the monitoring data and the optimal simulated value.

Firstly, based on the requirements of SWAT data format, the land-use database, soil database and meteorological database of the study area were reconstructed [29,42]. Second, based on DEM and hydrological data, the minimum area of sub-basin units was set in the SWAT model, the sub-basin units are delimited, and the surface runoff was simulated by inputting them into various databases. Third, the SWAT-CUP model and the measured data of a hydrological station were selected to verify the surface runoff simulation results of the SWAT model and calibrate the parameters. In the verification process, the percentage of bias (PBIAS, which measures the average trend between simulated and observed values), correlation coefficient (R^2^) and efficiency coefficient (Nash–Suttcliffe, or NS for short) [22] were used to evaluate the model verification results, and the relevant parameters were calibrated. Finally, based on the calibrated parameters, more accurate surface runoff values were obtained by re-simulation. The formula of PBIAS (unit: %) is as follows [22].

(1) $$\mathrm{PBIAS}=\frac{\sum_{j=1}^{n}\left(Q_{p,j}-Q_{o,j}\right)}{100\sum_{j=1}^{n}Q_{o,j}}$$

The calculation formula of correlation coefficient R^2^ (unitless) is as follows [22].

(2) $$R^{2}=\frac{{\left[\sum_{j=1}^{n}\left(Q_{o,j}-Q_{0,\mathrm{avg}}\right)\left(Q_{p,j}-Q_{p,\mathrm{avg}\;}\right)\right]}^{2}}{\sum_{j=1}^{n}{\left(Q_{o,j}-Q_{o,\mathrm{avg}}\right)}^{2}\sum_{j=1}^{n}{\left(Q_{p,j}-Q_{p,\mathrm{avg}}\right)}^{2}}$$

The calculation formula of efficiency coefficient NS (unitless) is as follows [22].

(3) $$\mathrm{NS}=1-\frac{\sum_{j=1}^{n}{\left(Q_{o,j}-Q_{p,j}\right)}^{2}}{\sum_{j=1}^{n}{\left(Q_{o,j}-Q_{o,\mathrm{avg}}\right)}^{2}}$$

$Q_{o,j}$ and $Q_{p,j}$ are the observed and simulated runoff (unit: m^3^) in time period *j*, respectively. $Q_{o,\mathrm{avg}}$ and $Q_{p,\mathrm{avg}}$ are the mean values of observed and simulated runoff (unit: m^3^), respectively. n indicates the timespan.

#### 3.2.2. Supply Capacity Measurement of FRES

The supply capacity of FRES in a sub-basin unit can be determined by the ratio of the hydrological regulation of the ecosystem in the unit to the rainfall [43]. Based on the original runoff depth and the actual runoff depth, both simulated by SWAT, the hydrological regulation of the sub-basin unit can be calculated.

(4) $$Q_{i}=\left(D_{bi}-D_{ai}\right)\times A_{i}\times 1000$$

$Q_{i}$ is the hydrological adjusting quantity of the ecosystem of sub-basin unit *i* (unit: m^3^). $D_{bi}$ is the bare land surface runoff depth of sub-basin unit *i* (unit: m). $D_{ai}$ is the actual surface runoff depth of sub-basin unit *i* (unit: m). $A_{i}$ is the area of sub-basin unit *i* (unit: km^2^).

(5) $$FRESsupply=\frac{Q_{i}}{P_{i}\times A_{i}\times 1000}\times 100\%$$

FRESsupply is the supply level of FRES of sub-basin unit *i* (unit: %). $P_{i}$ is the rainfall intensity of sub-basin unit *i* in a certain return period (unit: m).

### 3.3. Methods for Assessing the Demand for FRES in Human Societies

#### 3.3.1. Construction of Demand Assessment Model for FRES

The demand for FRES is closely related to the risk of flood damage. The higher the risk level, the stronger the demand for FRES. The risk level of flood damage is affected by the hazard of disaster-causing factors, the sensitivity of the disaster-prone environment and the vulnerability characteristics of disaster-bearing bodies [44,45]. Among them, the hazard of disaster-causing factors mainly derives from rainfall intensity and surface water distribution [39,45]. The sensitivity of the disaster-prone environment is mainly related to the physical characteristics of the underlying surface, such as surface slope, elevation, vegetation cover, soil type, and land use [45,46]. The vulnerability of the disaster-bearing body mainly involves the exposure degree and adaptability. The exposure of disaster-bearing bodies mainly includes population, economy, physical space, and facilities [39,46], while the adaptability of disaster-bearing bodies is related to disaster reduction and relief factors such as transportation channels, drainage network facilities, rescue facilities, and medical facilities [47]. Therefore, the demand for FRES can be calculated by model (6)–model (10).

(6) $$FRESdemand=\left(H\times W_{h}\right)\left(S\times W_{s}\right)\left(E\times W_{e}\right)/\left(A\times W_{a}\right)$$

FRESdemand is the demand index of FRES (unitless). H is the index of hazard of disaster-causing factors. S is the index of sensitivity of the disaster-prone environment. E is the index of exposure of the disaster-bearing body. A is the index of adaptability of the disaster-bearing body. $W_{h}$, $W_{s}$, $W_{e}$ and $W_{a}$ are the weights of the above indices, respectively.

(7) $$H=\sum_{1}^{i}H_{i}W_{hi}$$

$H_{i}$ is the standardized value of hazard index of disaster-causing factors such as rainfall intensity and surface water distribution (unitless). i is the number of the hazard index of disaster-causing factors. $W_{hi}$ is the corresponding weight of each indicator.

(8) $$S=\sum_{1}^{i}S_{i}W_{si}$$

$S_{i}$ is the standardized value of sensitivity index of the disaster-prone environment such as terrain, soil, vegetation, and land use (unitless). i is the number of the sensitivity index of disaster-prone environments. $W_{si}$ is the corresponding weight of each indicator.

(9) $$E=\sum_{1}^{i}E_{i}W_{ei}$$

$E_{i}$ is the standardized value of the exposure index of the disaster-bearing body such as GDP, population and cultivated land (unitless). i is the number of the exposure index of the disaster-bearing body. $W_{ei}$ is the corresponding weight of each indicator.

(10) $$A=\sum_{1}^{i}A_{i}W_{ai}$$

$A_{i}$ is the standardized value of the adaptability index of the disaster-bearing body such as emergency rescue facilities, roads, and drainage facilities (unitless). i is the number of the adaptability index of the disaster-bearing body. $W_{ai}$ is the corresponding weight of each indicator.

#### 3.3.2. Index Selection and Weight Calculation of FRES Demand Assessment

Based on the above analysis, this study selected assessment indicators to measure the social demand for FRES from four aspects: hazard of disaster-causing factors, sensitivity of disaster-prone environments, exposure of the disaster-bearing body, and adaptability of the disaster-bearing body. The names and meanings of each indicator are shown in Table 2.

We used the AHP method and entropy weight method to calculate the index weight. Firstly, the range normalization method was adopted to make the data dimensionless. Then the AHP method and entropy weight method were used to calculate the weight value of the index, respectively. Finally, the distance function and linear combination method were used to obtain the comprehensive weight value of each index [48].

The steps to determine the weight of indicators using the AHP are as follows. First, five experts in the field of flood disaster research from relevant research institutions distribute the weight evaluation table of FRES demand assessment index. In the table, each expert is asked to compare the importance of indicators at all levels of flood disaster risk in pairs, and the judgment scale is 1–9. Then, weight opinions are collected for pairwise comparison of each indicator. Based on YAAHP platform, the hierarchical structure model is constructed, and the expert score of pairwise comparison of indicators is used as the calculation basis to establish a pair judgment matrix of indicators at all levels. Finally, after calculation, the consistency ratio (CR) values of the index judgment matrix of the target layer, criterion layer, and indicator layer are all lower than 0.1, indicating that the calculated results of the index weight value pass the consistency test.

The formula for calculating the comprehensive weight is as follows.

(11) $$W_{i}{=\alpha W}_{i\;}^{\prime }{+\beta W}_{i}^{\prime \prime }$$

The calculation formula of the distance function is as follows.

(12) $$d\left(W_{i}^{\prime }{,W}_{i}^{\prime \prime }\right)={\left[\frac{1}{2}\overset{n}{\underset{i=1}{\sum }}{\left(W_{i}-W_{i}^{\prime }\right)}^{2}\right]}^{\frac{1}{2}}$$

The equations of distance function and weight distribution coefficient are as follows.

(13) $$\{\begin{array}{c} d{\left(W_{i}^{\prime }{,W}_{i}^{\prime \prime }\right)}^{2}=(\alpha -{\beta )}^{2}\\ \alpha +\beta =1 \end{array}$$

$W_{i}$ is the composite weight value. $W_{i}^{\prime }$ is the weight value calculated by the AHP method. $W_{i}^{\prime \prime }$ is the weight value calculated by the entropy weight method. α and β are the distribution coefficients of weights. The absolute difference between α and β is the difference between the distribution coefficients.

### 3.4. Methods for Comparing FRES Supply and Demand across Spatial Scales

#### 3.4.1. Comparison of FRES Supply and Demand at Macroscopic Watershed Scale

Based on the principle of social-ecosystem ES supply and demand, the FRES demand in each land patch can be mapped to its sub-basin units, and the relationship between the supply and demand of FRES can be compared at the macro-scale. The FRES demand of each land patch in the sub-basin unit is pooled, and the FRES demand is calculated by taking the sub-basin as the unit. The formula is as follows.

(14) $$FRESdemand_{j}=\frac{\sum_{i=1}^{n}FRESdemand_{\left(i,j\right)}\times A_{\left(i,j\right)}}{A_{j}}$$

$FRESdemand_{j}$ is the FRES demand of the sub-basin unit j (unitless). $FRESdemand_{\left(i,j\right)}$ is the FRES demand of the ith land patch in the sub-basin unit j (unitless). $A_{\left(i,j\right)}$ is the area of the ith land patch. n is the number of land patches in the sub-basin unit j (unit: km^2^). $A_{j}$ is the total area of the sub-basin unit j (unit: km^2^).

Then, we can compare the FRES supply and demand in sub-basins at the macroscopic watershed scale. Using the quantile method, each sub-basin is divided into high-supply-type and low-supply-type units according to the level of FRES supply, and into high-demand-type and low-demand-type units according to the level of FRES demand. Through spatial superposition processing, four types of regions are identified: “low supply–high demand”, “high supply–high demand”, “low supply–low demand”, and “high supply–low demand” [15]. Then, the ecological space scope that urgently needs to be protected and restored in each sub-basin unit is extracted at the macro-scale and, from the perspective of improving FRES, it provides a scientific basis for the zoning management of ecological space in response to flood damage.

#### 3.4.2. Comparison of FRES Supply and Demand with Land Patch as Unit at Micro-Scale

Based on the social-ecosystem ES supply and demand principle, the FRES supply of sub-basin units is decomposed to each land patch in the watershed, and the relationship between the supply and demand of FRES is compared at the micro-scale with land patch as the unit. The ratio of FRES supply and demand of each land patch is calculated as follows [49].

(15) $$SDR_{\left(i,j\right)}=\frac{A_{\left(i,j\right)}}{A_{j}}\times FRESsupply_{j}/FRESdemand_{\left(i,j\right)}$$

$SDR_{\left(i,j\right)}$ is the ratio of FRES supply and demand of the ith land patch in sub-basin unit j (unit: %). $A_{j}$ is the total area of the sub-basin unit j (unit: km^2^). $A_{\left(i,j\right)}$ is the area of the ith land patch (unit: km^2^). $FRESsupply_{j}$ is the FRES supply of the sub-basin unit j (unit: %). $FRESdemand_{\left(i,j\right)}$ is the FRES demand of the ith parcel in the sub-basin unit j (unitless).

The higher the ratio of FRES supply and demand, the stronger the flood-bearing resilience. The lower the ratio of FRES supply and demand, the greater the risk of flood, so the flood control and drainage planning measures should be further strengthened. This provides a basis for the zoning of flood control and the formulation of corresponding management strategies at the micro-scale of land patches.

## 4. Results

### 4.1. The Supply of FRES in Fujian Delta

**Figure 3:**
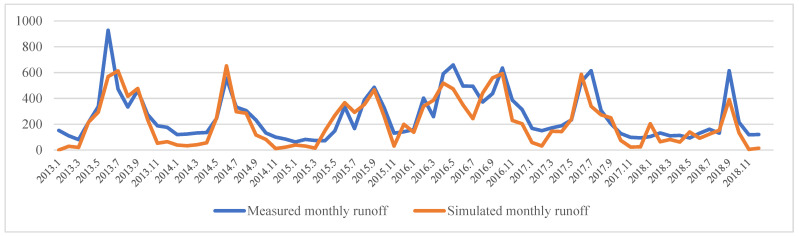
Comparison of measured and simulated monthly runoff.

Simulation values of surface runoff based on SWAT model are compared with monthly observation data of a hydrological station in Zhangzhou City, Fujian Province, and model parameters are verified (Figure 3). The study sets the warm-up period as 2011–2012, the rate period as 2013–2015, the validation period as 2016–2018, and the serial number of the verified sub-basin as 153. The results after parameter correction show that the correlation coefficient R^2^ is greater than 0.6, the efficiency coefficient NS is greater than 0.5, and the percentage deviation PBIAS is less than 25% (Table 3), indicating that the simulation effect of the model is good, and the simulation results can be used for further research.

**Figure 4:**
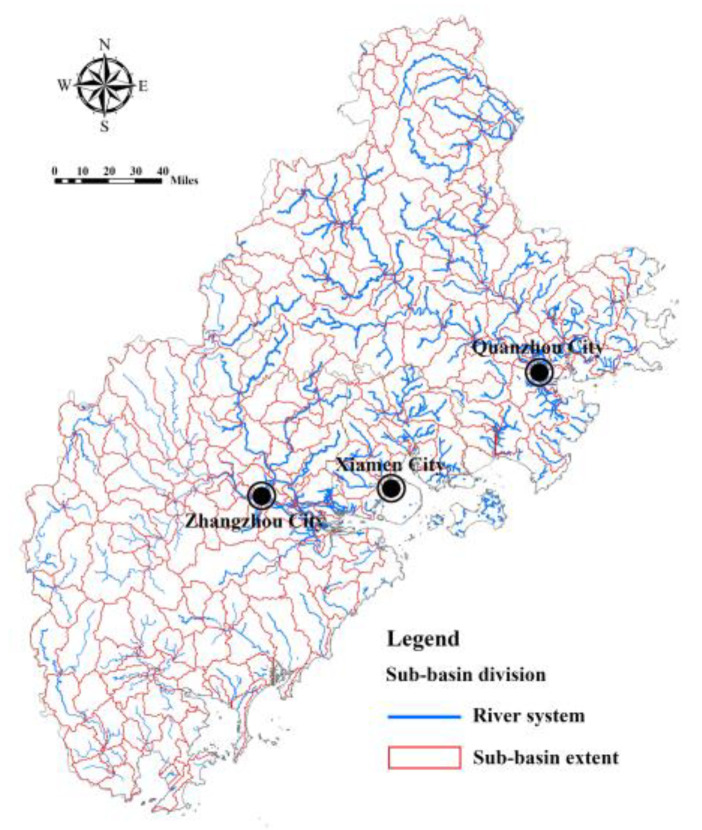
Drainage system distribution and sub-basin division in Fujian Delta.

**Figure 5:**
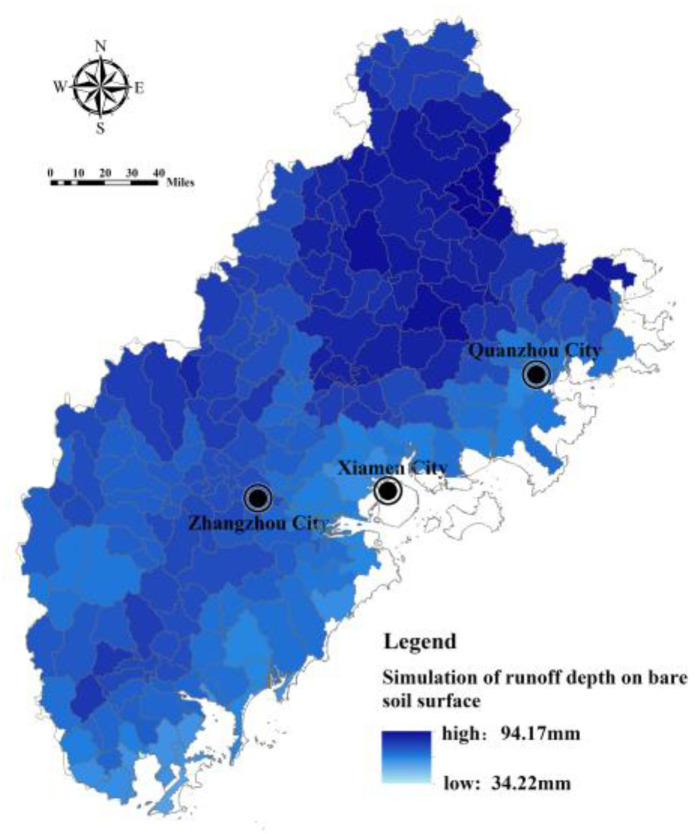
Runoff simulation of bare land surface in Fujian Delta.

**Figure 6:**
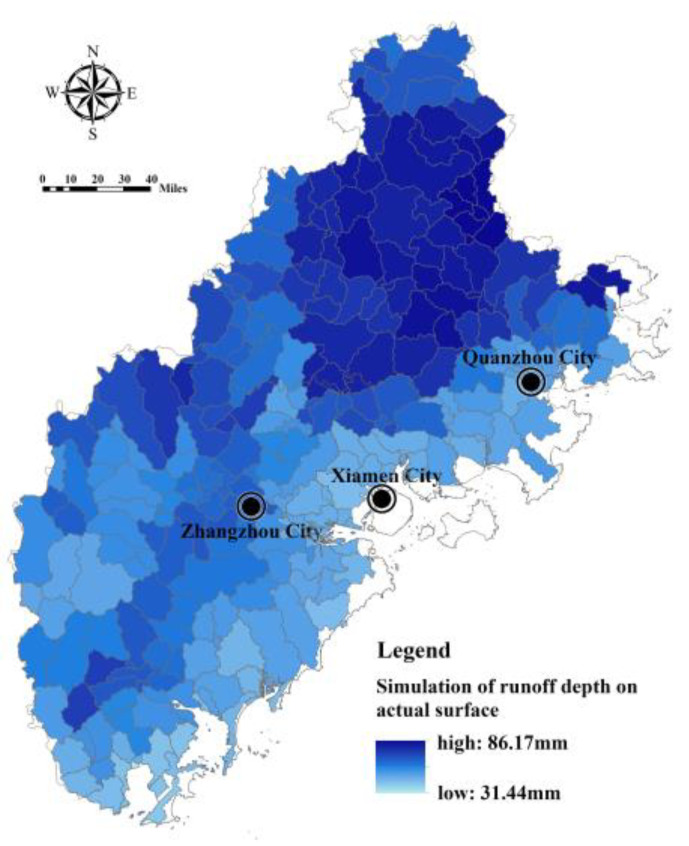
Simulation of actual surface runoff in Fujian Delta.

Based on the SWAT model, the Fujian Delta region is divided into 264 sub-basins, covering an area of 24,087 km^2^ (Figure 4). The bare land surface runoff depth and actual surface runoff depth are simulated by taking the sub-basin as a unit, and the results are shown in Figure 5 and Figure 6. The surface runoff depth of different sub-basin units in the Fujian Delta region varies greatly (the actual runoff depth varies by, at most, 2.74 times). The sub-basin units with a greater surface runoff depth are concentrated in the northern and western mountains. Meanwhile, the surface runoff depth of bare land in each sub-basin is generally greater than the actual surface runoff depth, indicating that the ecosystem plays a significant role in regulating regional hydrology.

**Figure 7:**
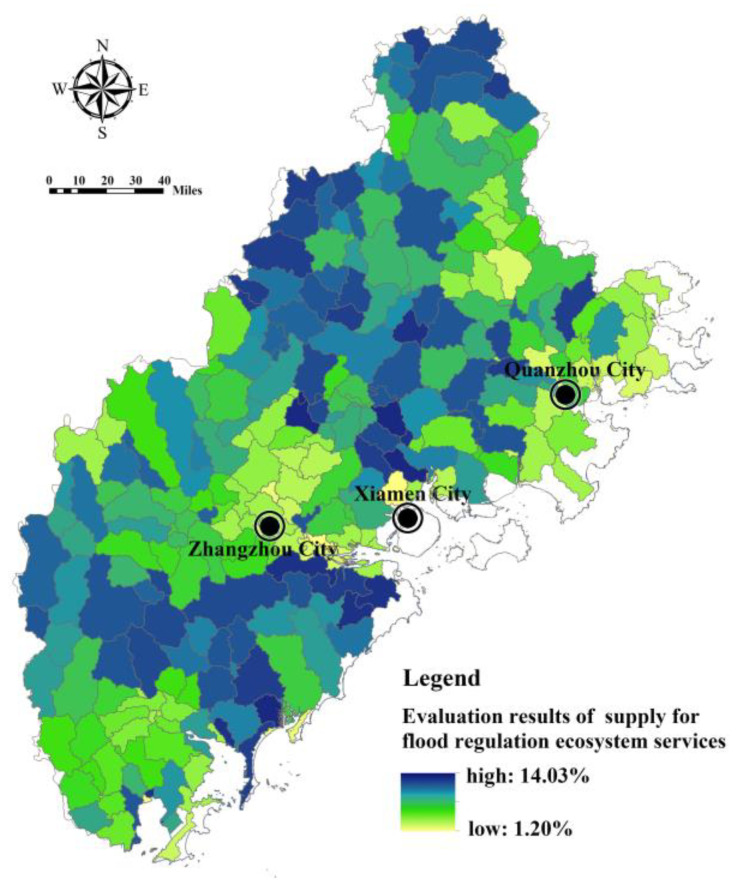
Assessment of FRES supply in Fujian Delta.

Based on the bare land surface runoff depth and the actual surface runoff depth simulated by SWAT, Models (4) and (5) are used to calculate the surface runoff regulation capacity of each sub-basin unit ecosystem in the Fujian Delta, namely, the supply level of FRES (Figure 7). It can be seen that the capacity of FRES varies greatly among the sub-basin units in the Fujian Delta (the difference between the maximum value and the minimum value is 11.69 times). The sub-basins with a higher supply of FRES are more widely distributed. The sub-basins with a low supply of FRES are mainly distributed in the dense urban areas of the lower reaches of the Jiulong River in Zhangzhou, Jimei urban area in Xiamen and Quanzhou coastal area. These sub-basin units are generally affected by urbanization, and their ecosystem is relatively fragile, so their FRES ability is low.

### 4.2. The Demand for FRES in Fujian Delta

**Figure 8:**
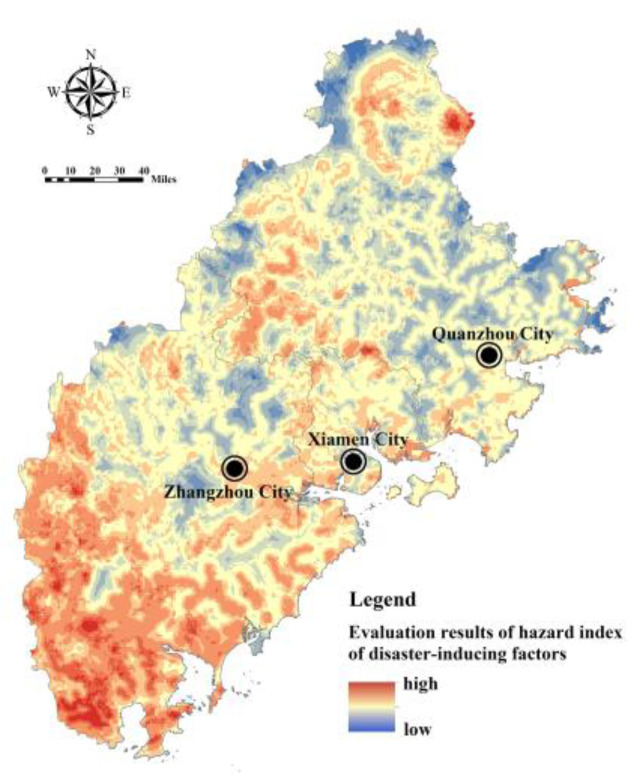
Assessment of the index of hazard of disaster-causing factors in Fujian Delta.

**Figure 9:**
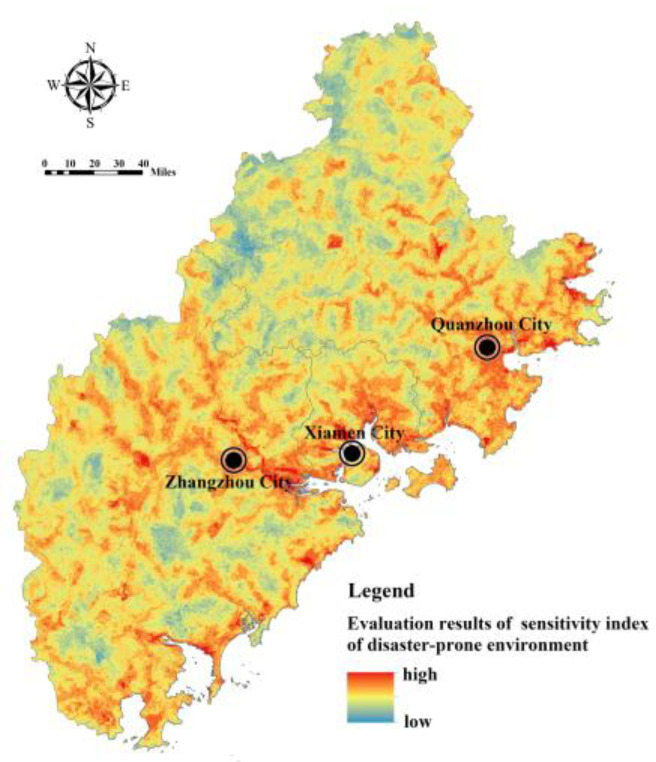
Assessment of the index of sensitivity of disaster-prone environments in Fujian Delta.

**Figure 10:**
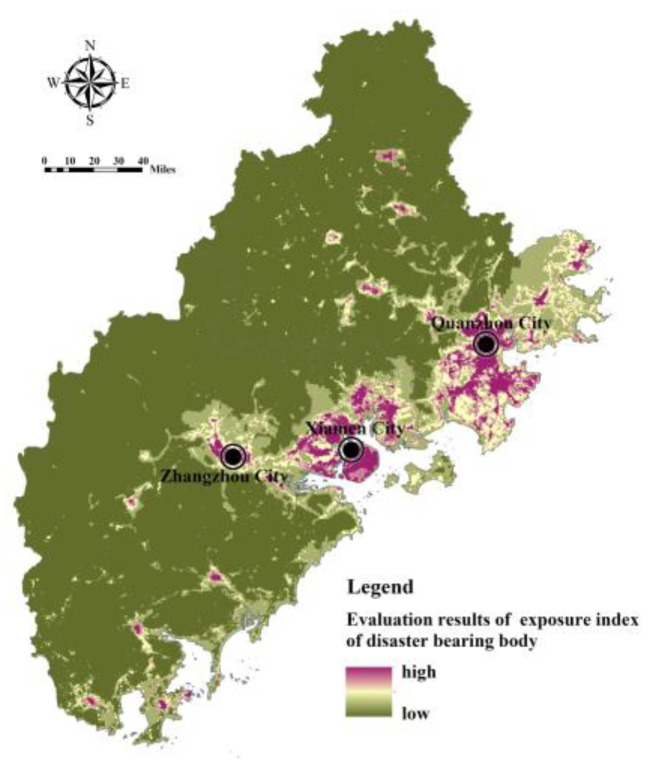
Assessment of the index of exposure of disaster-bearing bodies in Fujian Delta.

**Figure 11:**
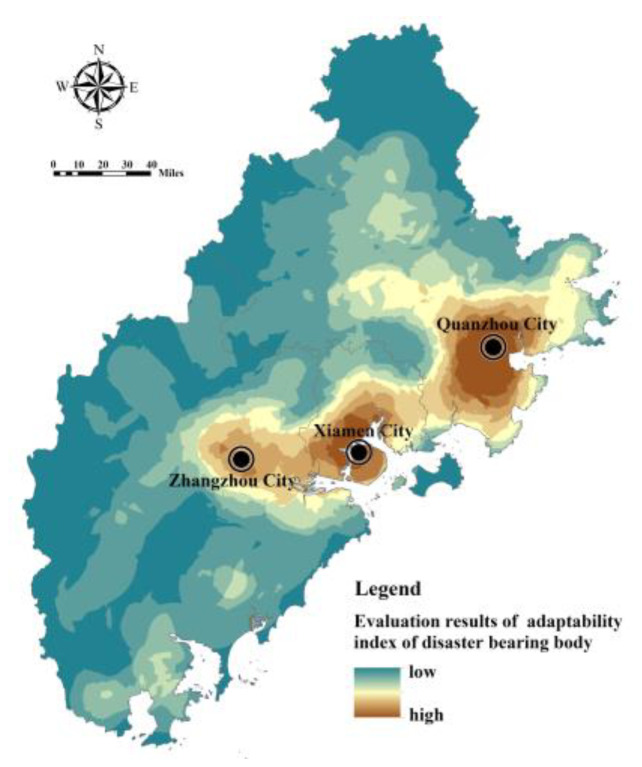
Assessment of the index of adaptability of disaster-bearing bodies in Fujian Delta.

Cronbach’s alpha coefficient is used to test the reliability level of the input data of the flood disaster risk assessment model [32]. According to the test, the Cronbach’s alpha coefficient value of the internal consistency reliability of flood disaster risk assessment index in Fujian Delta is 0.731, which met the requirements of index characterization and data consistency. Based on the index system of the FRES demand assessment, Model (7) and Model (10) were used to calculate the index of hazard of disaster-causing factors, the index of sensitivity of disaster-prone environment, the index of exposure of the disaster-bearing body, and the index of adaptability of the disaster-bearing body in Fujian Delta. The index of hazard of disaster-causing factors is affected by the rainfall intensity and buffer area of the water area, showing an overall pattern of being high in the southwest and low in the northeast (Figure 8). The index of sensitivity of the disaster-prone environment is comprehensively affected by topography, soil, vegetation, and land use. The regions with high sensitivity are mostly distributed in river valleys, coastal zones, and areas with high construction intensity, low vegetation coverage, and low-lying terrain (Figure 9). The index of exposure of the disaster-bearing body is affected by the density of populations, economies, and facilities. The areas with high exposure are highly correlated with the distribution of urban production and living space, being concentrated in high-density built-up areas of Xiamen, Zhangzhou and Quanzhou and some township resident areas (Figure 10). The adaptability index of the rainfall–flood disaster-bearing body is affected by drainage facilities, rescue facilities, medical facilities, and traffic facilities, and the areas with strong adaptability are mainly distributed in the urban areas and the extension areas of the surrounding traffic corridors (Figure 11).

**Figure 12:**
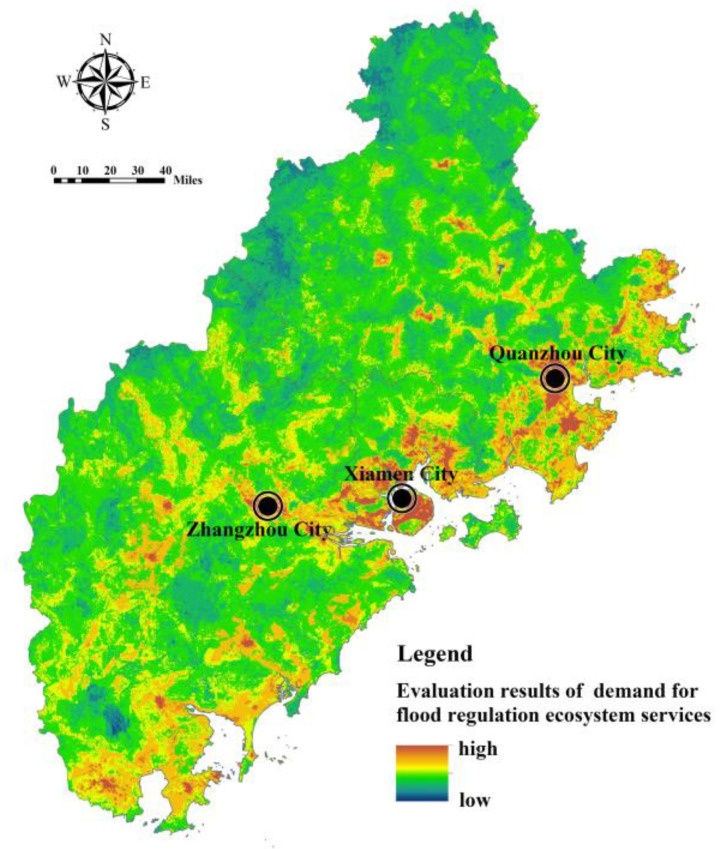
Assessment of the demand for FRES in Fujian Delta.

Model (6) was used to calculate the demand index for FRES in the Fujian Delta (Figure 12). It can be seen that the areas with high demand for FRES are concentrated in high-density urban built-up areas, parts of the river valleys, and coastal zones. River valleys and coastal areas have a high risk of being inundated by rising water levels due to their low-lying terrain and proximity to water areas, and the distribution of urban and rural construction land and cultivated land is relatively concentrated. Therefore, the demand for FRES is high. Despite the dense distribution of drainage, rescue, medical care, and transportation facilities in towns and their surrounding areas and their relatively strong ability to cope with flood disasters, high-density urban areas still have the highest demand for FRES compared with ecological and agricultural spaces. However, within the built-up area, the distribution of disaster reduction and relief facilities has a certain impact on the spatial difference in the demand for FRES, which can provide a basis for the hierarchical layout of flood control projects and relief facilities.

### 4.3. Comparison of FRES Supply and Demand across Spatial Scales

#### 4.3.1. Macroscopic FRES Supply and Demand Comparison and Zoning Management of Ecological Space in Fujian Delta

**Figure 13:**
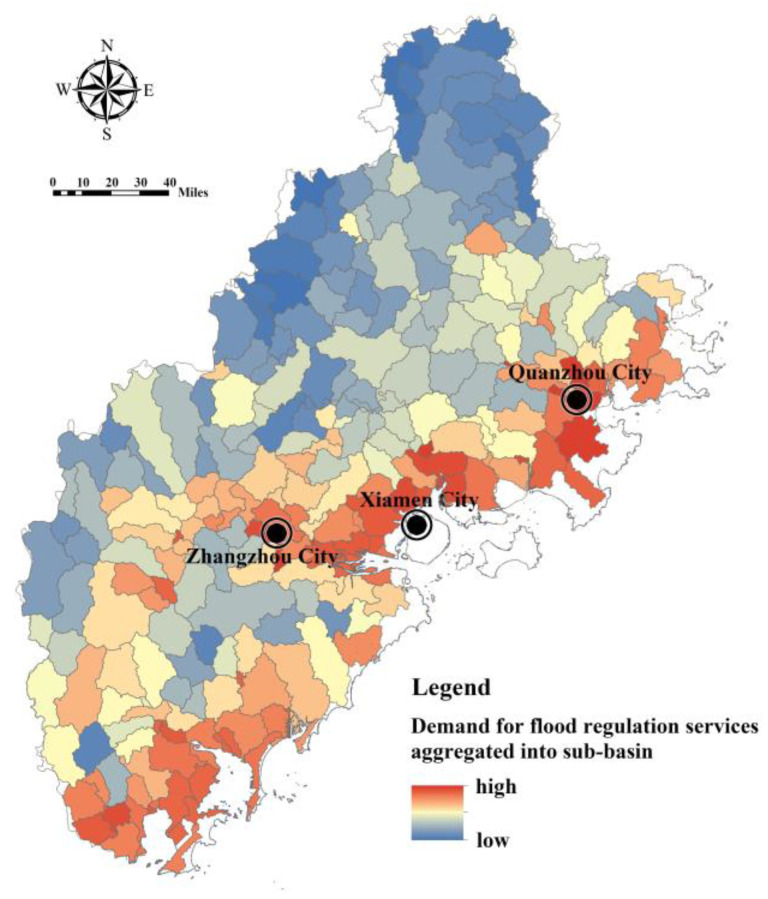
The demand of FRES converging to sub-basins of Fujian Delta.

We compared the supply and demand of stormwater regulation services at the macro-scale by taking the sub-basin as the unit and identifying the sub-basin units with an imbalance in supply and demand, to provide a basis for the ecological spatial zoning management plan to cope with rain and flood risk. Model (14) was used to calculate the sum of the FRES demands of all land patches in each sub-basin unit (Figure 13). It can be seen that the sub-basin units with a higher demand for FRES are mainly distributed in the coastal zone and Zhangzhou Jiulong River valley, due to the low-lying terrain, dense population and the distribution of construction land.

**Figure 14:**
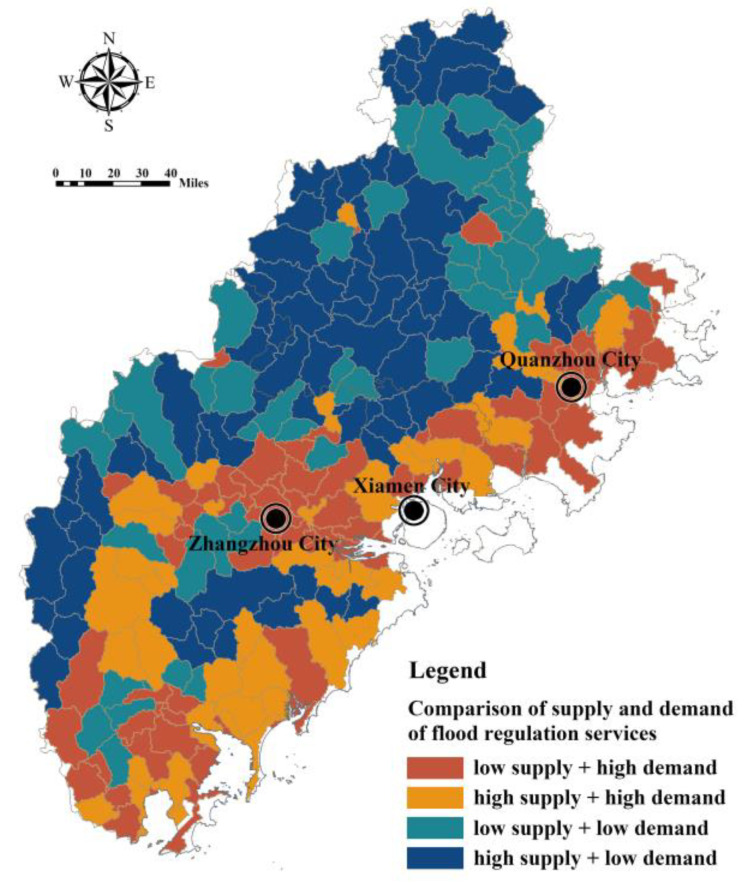
Comparison of FRES supply and demand at the sub-basin level in Fujian Delta.

The sub-basin was taken as a unit to compare the relationship between supply and demand of FRES in Fujian Delta, and four sub-basin units were identified, namely, low supply–high demand, high supply–high demand, low supply–low demand and high supply–low demand (Figure 14). Among them, the contradiction between supply and demand of low supply–high demand units was the most prominent; this is an area that urgently needs to protect and restore ecological space to improve the supply of FRES. There are 71 sub-basin units of low supply–high demand in the Fujian Delta, mainly distributed in the southeast coast and the lower reaches of Jiulong River. The demand for FRES in high supply–high demand units is high, so it is necessary to maintain a FRES high supply level, and its ecological space should be properly protected and restored. The demand for FRES in low supply–low demand units is low. However, considering the possible FRES demand growth caused by urbanization in the future, the ecological space should be properly protected to ensure the supply of FRES.

**Figure 15:**
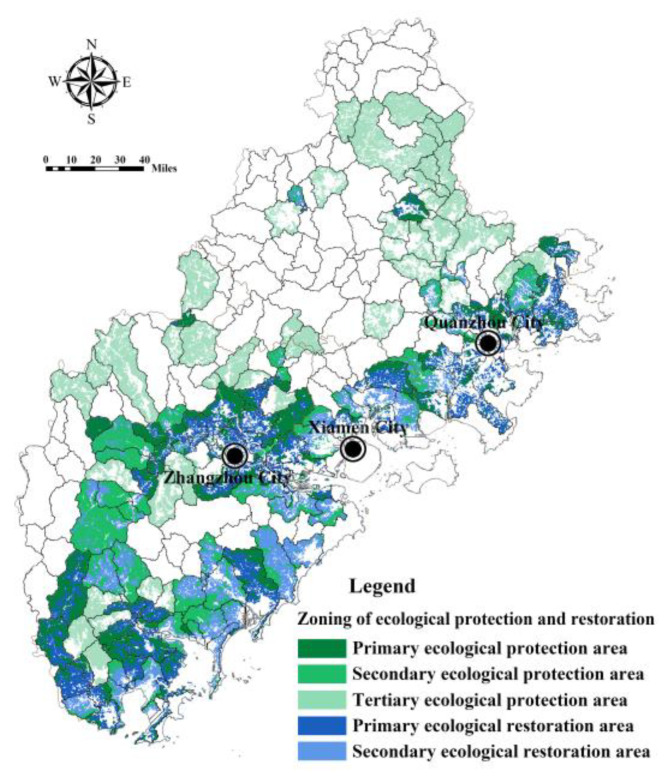
Zoning management of ecological space in Fujian Delta based on the comparison of FRES supply and demand in the sub-basin.

Based on the above analysis, from the perspective of coping with flood risk, the ecological space (woodland, grassland, wetland) in each sub-basin unit was divided into primary, secondary, and tertiary ecological protection areas and non-ecological protected areas. The space required for ecological restoration (cultivated land and bare land) was divided into primary, secondary ecological restoration areas, and non-ecological restoration areas (Table 4). Among them, the ecological space in the low supply–high demand sub-basin was the primary ecological protection area, and the most stringent ecological protection measures were implemented. The cultivated land and bare land in the sub-basin were the primary ecological restoration areas, and conversion of farmland to forest and vegetation restoration projects will be implemented. The ecological space in the high demand–high supply sub-basin was the secondary ecological protection area, and the cultivated land and bare land in the sub-basin were the secondary ecological restoration areas. The ecological space in the sub-basin of low demand–low supply was the tertiary ecological protection area. There was no ecological protection area and ecological restoration area in the high supply–low demand sub-basin. Figure 15 shows the zoning management plan of ecological space in Fujian Delta used to cope with flood risk. It can be seen that the primary ecological protection areas and ecological restoration areas were mainly located in the coastal and Zhangzhou Jiulong River downstream areas. The secondary and tertiary ecological protection areas were mostly located in the middle and upper reaches of rivers.

#### 4.3.2. Microscale FRES Supply and Demand Comparison and Flood Control Zoning Intervention in Built-Up Areas in Fujian Delta

**Figure 16:**
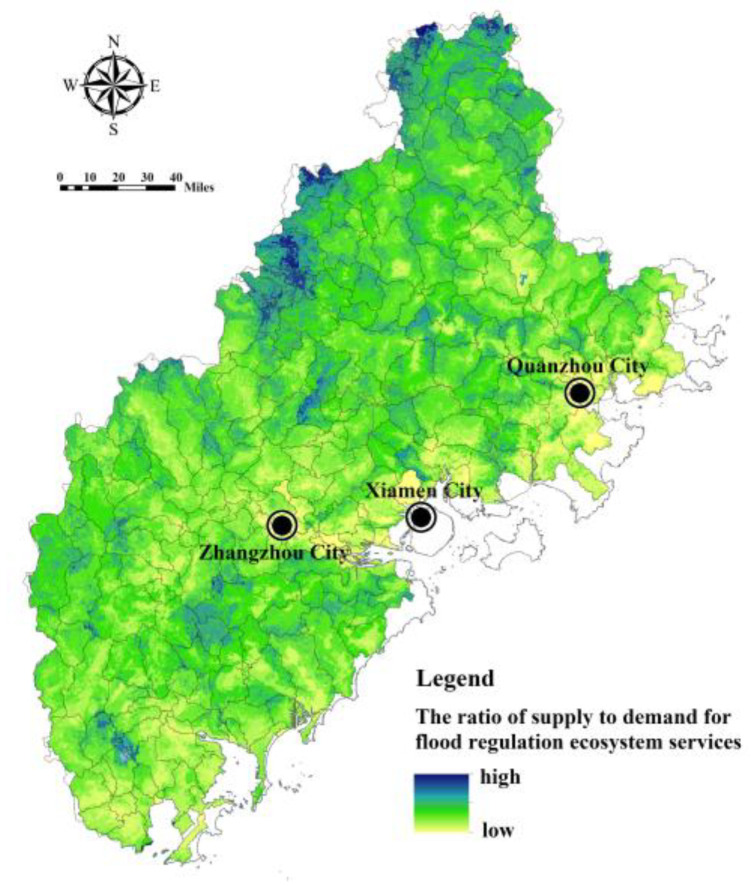
Analysis of the ratio between FRES supply and demand in Fujian Delta based on land patch.

Based on land patches, the micro-scale ratio of FRES supply and demand was calculated, and the land patch units with an imbalance between supply and demand were identified to provide a basis for formulating the zoning intervention plan of built-up areas to cope with flood risk. Model (15) was used to decompose the FRES supply of the sub-basin into each land patch, and the ratio of FRES supply and demand in the land-patch unit was calculated (Figure 16). The natural discontinuous point method was used for classification research [39]. For example, the number of land patches with the lowest ratio of FRES supply and demand is 13,257, with a total area of 132.57 km^2^. These land patches are mainly distributed in the coastal areas of Xiamen, Quanzhou and the middle and lower reaches of Zhangzhou Jiulong River Valley. Due to the imbalance in FRES supply and demand, it is urgent to improve flood disaster adaptability through flood control projects.

**Figure 17:**
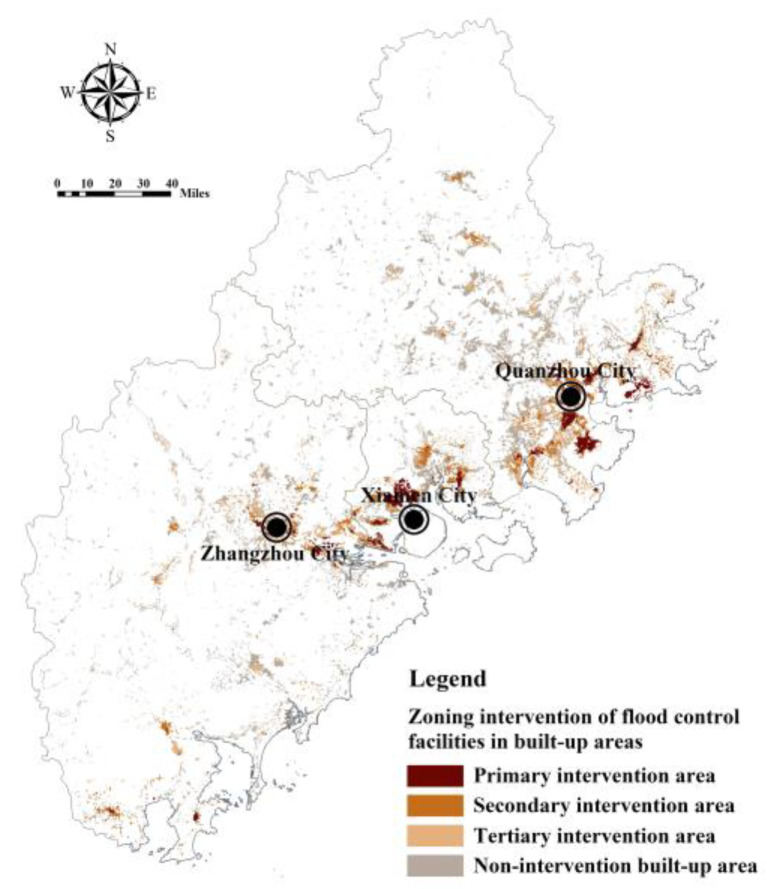
Regional intervention of flood control facilities in built-up areas of Fujian Delta based on a comparison of FRES supply and demand.

In the regions with a low ratio of FRES supply and demand, the urban and rural construction land patches were extracted and used as the specific scope of flood control facilities construction in the Fujian Delta. According to the classification results of the ratio of FRES supply and demand, the areas with a low ratio were divided into the lowest type, the lower type, and the slightly lower type, and the construction land patches within the scope correspond to the primary, secondary, and tertiary flood control project intervention areas, respectively (Table 5). For example, the area with the lowest ratio of FRES supply and demand is 132.57 km^2^, and the construction land area within its scope is 65.42 km^2^, involving Quanzhou Fengze cluster, Jinjiang and Shishi Center cluster, Xiamen Jimei Houxi cluster, and Zhangzhou Xiangcheng cluster which is divided into the primary intervention area (Figure 17). In the future, this region should carry out high-precision inundation risk area calculations based on a detailed urban drainage network and terrain data [15], to accurately lay out flood control engineering facilities and disaster relief facilities and improve urban flood resilience.

## 5. Discussion

The main contribution of this study is to construct a cross-spatial framework for comparing supply and demand of regional FRES based on the concept of “service spatial flow” from a socio-ecosystem perspective. On the one hand, the FRES demand of land patches was collected to the sub-basin unit, and the sub-basin unit with an imbalance in the supply and demand of FRES was identified, so the ecological space that needs to be protected and the non-ecological space that needs to be repaired in the unit were extracted. On the other hand, the FRES supply of the sub-basin was decomposed into the land-patch units, so the land patches with an imbalance between the supply and demand of FRES were identified, and the built-up areas that urgently need flood control project intervention were extracted. In the application of the technology, the SWAT model and the SWAT-CUP calibration model were used to simulate the real hydrological formation process in a complex environment which enhanced the reliability of the FRES supply’s measurement results. In the flood risk assessment index system, the index of adaptability of the disaster-bearing body reflecting the disaster reduction and relief ability was added, which improves the accuracy of the FRES demand measurement. Regional spatial governance schemes such as ecological protection zoning, ecological restoration zoning, and flood control intervention zoning to cope with flood risk were formed in this study, which improves the application of such research results in practice.

The SWAT-CUP verification results of surface runoff simulation in Fujian Delta show that the simulated values were in good agreement with the actual measured values of hydrological stations, and the simulation effect of the SWAT model was good, which can better reflect the regional hydrological formation process. Therefore, the FRES supply results in the Fujian Delta, calculated based on surface runoff, have high reliability. Based on SWAT-CUP, we verified the simulated values of surface runoff using R^2^, NS and PBIAS. In fact, there are many verification methods for surface runoff simulation [29,42]. For example, the comparison of runoff images based on remote sensing images is also valuable [15]. Therefore, a variety of calibration methods can be used in the future to improve the accuracy of surface runoff simulation.

In the flood risk assessment, the index of inundation scope was adopted in relevant studies to assess the hazard of disaster-causing factors [39]. However, considering that the terrain, rainfall, vegetation, and land use used in the simulation of inundation scope are highly repetitive with other indicators in the risk assessment index system of this paper, we did not adopt this index. In terms of sensitivity assessment, the relevant research adopts random forest or BP neural network methods, conducts training and testing through the water accumulation data of some experimental samples and multi-factor datasets, determines the model parameters or nonlinear relations, and finally injects the original data into the model to realize the global prediction [38,50]. These methods are very effective in solving the problem of data shortage [38], but because the basic data of this study can cover the whole range of Fujian Delta, we did not adopt the above methods. Although the index of adaptability of the disaster-bearing body reflecting disaster reduction and relief ability was added, the demand for FRES in urban built-up areas in Fujian Delta was still significantly higher than that in ecological and agricultural space but makes a contribution to distinguishing the spatial differences in internal demand in built-up areas, which provides a basis for delimiting flood control intervention zones. In the comparison of ES supply and demand, another common calculation method was adopted in relevant studies, that is, the difference between supply and demand was divided by half of the maximum sum of supply and demand [11]. However, the dimensional difference between FRES supply and demand in this study was large, so we chose the more suitable direct ratio method.

At present, the practical application research in this paper is mainly focused on the optimization of FRES capacity in sub-basins. For example, from the perspective of coping with flood risk, the zoning scheme of ecological space protection, ecological restoration, and flood control intervention is proposed. However, less consideration was given to ecosystem protection measures in the upper reaches of the sub-basin. In fact, the upstream ecosystem contributes to the regulation of surface runoff in the downstream sub-basin [1,22], which is described in the application principle of the SWAT model in this paper. Therefore, in addition to identifying and protecting the ecological space in the sub-basin with imbalances between supply and demand, future studies can also identify the ecological space in the upper reaches of the sub-basin and propose specific protection and restoration measures [1]. This leads to another logic of the prioritization of ecological protection and restoration. For example, high priority can also be given to areas where water resources can be maximized for conservation. Although these FRES high-supply areas have a low flood risk, they are located in the upper reaches of the catchment and, through water conservation and vegetation protection, can provide more FRES to the downstream sub-basin. The implementation difficulty of ecological restoration is also less than that of highly urbanized sub-basins. Therefore, it is a new idea to define the ecological protection and restoration area in the future by combining the logic of high supply sub-basin priority and unbalanced supply sub-basin priority.

In this study, due to the incomplete overlap between the administrative boundary and the watershed boundary and the complex distribution of coastal catchment areas, the sub-basin division results based on the SWAT model did not cover the entire Fujian Delta, and 4.88% of the regions were not included in the comparative study of FRES supply and demand. In the future, the factor and weight of surface runoff regulation rate can be calculated by the neural network algorithm [38,39], and the FRES supply level in the previously uncovered area can be calculated, so that the research results can cover the whole area. In addition, the high-demand spaces and flood control intervention areas identified through the study of the supply and demand of regional FRES, cannot replace the refined risk study of flood damage in urban areas. It can, however, provide a reference for further calculation of flood inundation areas in urban areas, and the layout of specific drainage engineering facilities and disaster relief facilities, and avoid blindness caused by too large a research scope.

## 6. Conclusions

By comparing the relationship between the supply and demand of regional FRES, this study draws a zoning management plan, which provides a decision-making basis for regional flood risk management. Compared with previous studies, this study has three advantages. Firstly, based on the socio-ecosystem perspective and applying the concept of “service spatial flow”, a cross-spatial scale comparison method of regional FRES supply and demand was proposed. This realizes the identification of ecological space in need of protection by sub-basins and the identification of construction land in need of flood control intervention by land patches. Second, the SWAT model and SWAT-CUP calibration model were used to simulate the real hydrological formation process in the regional environment, and the index of adaptability of the disaster-bearing body was added to the storm flood risk assessment, which enhanced the accuracy of the measurement results of FRES supply and demand. Thirdly, the zoning schemes of ecological space protections, ecological restorations and flood control interventions were proposed to deal with flood risk, which improved the application of our research results in spatial management practices.

Taking Fujian Delta as an example, the study shows that the FRES supply varies greatly among the sub-basins, and the sub-basins with a low supply are mostly distributed in the lower reaches of Jiulong River, coastal areas, and other low-lying areas that are strongly affected by urbanization. The demand for FRES in high-density urban built-up areas is much higher than that in ecological and agricultural spaces. Adding the index of adaptability of the disaster-bearing body to the demand assessment of FRES can help to distinguish the spatial differences in internal demand in built-up areas and identify the land patches that most need flood control interventions. The 2153 km^2^ ecological spaces of 71 “low supply–high demand” sub-basin units were classified as the primary ecological protection areas, and 914 km^2^ of cultivated land and bare land were classified as the primary ecological restoration areas by pooling the FRES demands of each land patch into sub-basin units and comparing the supply and demand of FRES. By allocating FRES supply of sub-basin to land-patch units, and comparing FRES supply and demand, 65.42 km^2^ construction land in 13,257 land-patch units with the lowest supply and demand ratio was classified as the primary intervention area. The above spatial regionalization results can be connected with the practical spatial management mechanism represented by territorial spatial planning and provide support for the spatial zoning management of flood risk from the perspective of FRES.

## Figures and Tables

**Table 1:** Main data characteristics and sources.

Data Name	Type	Characteristic	Sources
NDVI	raster data	In 2020/1 km × 1 km	U.S. Geological Survey Landsat image (https://earthexplorer.usgs.gov, accessed on 15 July 2022)
Medical and firefighting facilities	vector data	In 2020	AMAP POI open data
Municipal drainage line	vector data	In 2020	Xiamen Urban Planning and Design Institute (non-public data)
DEM	raster data	30 m × 30 m	Geospatial data cloud (https://www.gscloud.cn/, accessed on 19 July 2022)
Land utilization	raster data	In 2020/30 m × 30 m	Data Center for Resources and Environmental Sciences, Chinese Academy of Sciences (https://www.resdc.cn/, accessed on 22 July 2022)
Soil type	raster data	1 km × 1 km	HWSD Soil Database (https://www.fao.org/soils-portal/en/, accessed on 17 July 2022)
Weather data	vector data	daily from 2008 to 2018	CMADS data set [42] (http://www.cmads.org/, accessed on 21 July 2022)
Hydrologic station data	table data	Monthly runoff from 2011 to 2021	A hydrological station in Zhangzhou City, Fujian Province (non-public data)
Water system and roads	vector data	In 2020	Universal electronic map of Shuijingzhu
Precipitation	raster data	1 km × 1 km	National Tibetan Plateau Environment Data Center (http://data.tpdc.ac.cn/zh-hans/, accessed on 15 July 2022)
The lights at night	raster data	In 2020/130 m × 130 m	Luojia No.1 data (http://59.175.109.173:8888/app/login.html, accessed on 15 July 2022)
Density of population	raster data	In 2020/100 m × 100 m	WorldPop Spatial Demographic Data and Research (https://www.worldpop.org/, accessed on 20 July 2022)
GDP	raster data	In 2019/1 km × 1 km	Resource and Environment Science and Data Center (https://www.resdc.cn/, accessed on 21 July 2022)

**Table 2:** Assessment index system of regional demand for FRES.

Category	Name of Index	Units	Implication
Hazard of disaster-causing factors	Rainfall intensity [39,45]	mm	The higher the rainfall, the higher the risk of flooding
	Buffer distance of water [39]	m	The greater the distance from the water, the lower the risk of inundation by rising river and lake levels
Sensitivity of disaster-prone environment	NDVI [24,39]	normalized value	The higher the vegetation coverage, the stronger the capacity of stormwater retention and the lower the risk
	Elevation [32,39]	m	The higher the land, the less vulnerable to flooding and the lower the risk
	Slope gradient [32,39]	%	The higher the slope of the land, the easier it is to discharge flood water, and the lower the risk
	Agrotype [46]	normalized value	The more permeable the soil, the less water on the surface and the lower the risk
	Land utilization [45,46]	normalized value	The higher the intensity of artificial construction, the higher the risk of surface ponding
Exposure of disaster-bearing body	Density of population [39,46]	Person/km^2^	The greater the population density, the more people that are potentially affected and the higher the risk
	Density of GDP [39,46]	Thousand yuan/km^2^	The higher the economic value, the greater the possible economic loss and the higher the risk
	Density of building [46]	m^2^/hm^2^	The greater the density of the building, the more facilities that can be damaged by storm water, and the higher the risk
	Cultivated area [45]	m^2^	The more arable land there is, the more serious the destruction of agricultural production may be, and the higher the risk
Adaptability of disaster-bearing body	Drainage network density [15]	km/km^2^	The more drainage network facilities, the stronger the municipal drainage capacity and disaster resilience
	Road density [47]	km/km^2^	The higher the road density, the safer the rescue and transportation channels and the stronger the disaster resilience
	Density of medical facilities [47]	number/km^2^	The more medical facilities that are nearby, the stronger the emergency rescue capacity and disaster resilience
	Density of fire and rescue facilities [47]	number/km^2^	The more fire and rescue facilities that are nearby, the stronger the emergency rescue ability and disaster adaptability

**Table 3:** Calibration results of SWAT surface runoff simulation based on measured data.

Phase Division	R^2^	NS	PBIAS/%
Verification Period (2013–2015)	0.76	0.68	15.0
Validation Period (2016–2018)	0.75	0.65	19.9

Note: R^2^ > 0.6, NS > 0.5 and PBIAS < 25% indicate that the simulation effect is good [22].

**Table 4:** Ecological spatial regionalization of the Fujian Delta based on a comparison of FRES supply and demand in the sub-basin.

Classification of Supply and Demand for FRES (Sub-Basin Units)	Ecological Space to Be Protected (Woodland, Grassland, Wetland)	Non-Ecological Space Requiring Ecological Restoration (Arable Land, Bare Land)
Low Supply–High Demand (71 units)	Primary ecological protection area (2153 km^2^)	Primary ecological restoration area (914 km^2^)
High Supply–High Demand (55 units)	Secondary ecological protection area (2028 km^2^)	Secondary ecological restoration area (1069 km^2^)
Low Supply–Low Demand (47 units)	Tertiary ecological protection area (3979 km^2^)	Non-ecological restoration area
High Supply–Low Demand (91 units)	Non-ecological protected area	

**Table 5:** Intervention zoning of flood control projects in the Fujian Triangle built-up area based on the ratio of FRES supply and demand in land patches.

Classification Based on Ratio of FRES Supply and Demand (Land Patch Unit)	Construction Land Zoning Requiring Flood Control Engineering Intervention	Flood Control Engineering Intervention Measures
The lowest type (13,257 units)	Primary intervention area (65.42 km^2^)	Elaborate identification of inundated areas and high standard layout of flood control and disaster relief facilities
The lower type (29,059 units)	Secondary intervention area (142.19 km^2^)	Identification of inundated areas and common standard layout facilities
Slightly lower type (60,036 units)	Tertiary intervention area (297.25 km^2^)	Ecological optimization and facility intervention as a supplement
Medium and high type	Built-up areas without flood control intervention	None

## Data Availability

The data presented in this study are available on request from the corresponding author.
